# Factors associated with home visits by volunteer community health workers to implement a home-fortification intervention in Bangladesh: a multilevel analysis

**DOI:** 10.1017/S1368980019003768

**Published:** 2021-04

**Authors:** Haribondhu Sarma, Md Tariqujjaman, Mduduzi NN Mbuya, Sufia Askari, Cathy Banwell, Thomas J Bossert, Catherine D’Este, Tahmeed Ahmed

**Affiliations:** 1 Nutrition and Clinical Services Division, icddr,b, Dhaka 1212, Bangladesh; 2 Research School of Population Health, The Australian National University, Acton, ACT 2601, Australia; 3 Global Alliance for Improved Nutrition, Dhaka, Bangladesh; 4 The Children’s Investment Fund Foundation, London, UK; 5 Harvard T.H. Chan School of Public Health, Boston, MA, USA; 6 School of Medicine and Public Health, Faculty of Health and Medicine, The University of Newcastle, Callaghan, NSW, Australia

**Keywords:** Community health worker, Home visit, *Sasthya Shebika*, BRAC, Home fortification

## Abstract

**Objective:**

BRAC, an international development organization based in Bangladesh, engages community health workers called *Shasthya Shebikas* (SS) to implement home fortification of foods with micronutrient powders (MNP). We identified factors associated with home visits by SS, at different levels of the BRAC programme-delivery hierarchy, to implement home-fortification interventions.

**Design:**

We conducted a cross-sectional survey, semi-structured interviews, and collected programme-related data from sub-districts included in the caregiver survey of BRAC’s home-fortification programme and performed multilevel logistic regression modelling to investigate factors associated with home visits by SS.

**Settings:**

Sixty-eight sub-districts in Bangladesh.

**Participants:**

Caregivers of children aged 6–59 months (*n* 1408) and BRAC’s SS (*n* 201).

**Results:**

Households with older children (0·55; 0·42, 0·72; *P* < 0·001) and located >300 m from the SS’s house (0·67; 0·50, 0·89; *P* = 0·006) were less likely to have been visited by the SS, whereas those with caregivers who had ≥5 years of schooling (1·53; 1·10, 2·12; *P* = 0·011) were more likely to have been visited by the SS (adjusted OR; 95 % CI). Households in the catchment area of older SS aged >50 years (0·44; 0·21, 0·90; *P* = 0·025) were less likely to have been visited by the SS, whereas those with SS who received incentives of >800 BDT (3·00; 1·58, 5·58; *P* = 0·001) were more likely to have been visited by the SS (adjusted OR; 95 % CI).

**Conclusions:**

The number of home visits is a function of the characteristics of SS, factors that characterize the households they serve and characteristics of their organizational context, particularly to implement home fortification of foods with MNP.

Community health workers (CHW) work on the front line, playing critical roles in addressing the shortage of health-related human resources in many low- and middle-income countries. Generally, they are community members who are usually chosen by a community-based organization or a local public health organization to provide basic health and medical care to their community. Since the CHW come from the same community in which they work, they have a sound understanding of the local culture, norms and community language. These skills enable them to freely access community members and better understand health problems from a local sociocultural context. CHW generally do not have a high level of technical expertise; however, most have primary-level education and training that enables them to read, write and perform simple mathematical calculations^(1)^. CHW with this expertise and education are usually paid, although some volunteer CHW, who are less likely to be literate, receive incentives either in monetary or non-monetary form instead of regular payment for their work.

CHW deliver a number of health and nutrition interventions, especially for children and women^(2,3)^. These include promotion of preventive interventions such as behaviour change interventions, promoting immunization, health education, and one-to-one counselling on hand washing, breast-feeding and complementary feeding; providing primary treatment for some common infectious diseases; and providing health screening and assessing nutritional status^(2,3)^. In delivering these interventions, the CHW, in general, perform multiple tasks^(4)^ where their first and main task is to visit a home and ensure the accessibility of their increased coverage of services to the target population of the community. Literature suggests that home visits by trained CHW improve health outcomes for sick newborns and young infants in resource-limited areas^(5–8)^. Despite positive effects of home contact by CHW, they face numerous challenges in performing their duties^(9)^ in many parts of the world, including limited resources, low motivation, inadequate rewards and incentives, lack of understanding of their work among community members, their own inadequate knowledge about their work, inadequate training, lack of supportive supervision, distrust from the community, and other challenges that impede their performance^(2,4,9–11)^.

## Community health workers in Bangladesh: a BRAC experience

BRAC, an international development organization based in Bangladesh, is a pioneer in using female volunteer CHW called *Sasthya Shebika* (SS)^(12)^. It is estimated that about 80 000 SS work in Bangladesh^(1)^. The SS are the core of BRAC’s community-based health interventions, serving as the first point of contact between communities and BRAC’s health and nutrition services. Each SS is responsible for 150–450 households. Most of the households allocated to an SS are situated in proximity of the SS’s home, so the SS can visit them all within a 2–3 h period. Earlier analysis suggested that on average an SS working 3.6 h could visit fourteen households per day^(13)^. On average SS receive 5 years of schooling; however, a quarter of SS are illiterate^(14)^. Most of BRAC’s SS do not have paid employment besides working as an SS, although a quarter of them are involved in agricultural work^(14)^.

After SS are recruited, BRAC provides them with a 3-week-long basic training on maternal, neonatal and child health, nutrition, immunization, family planning, and a few common diseases such as the common cold, fever, cough, diarrhoea, anaemia, worm infection and scabies^(15)^. Additionally, SS also receive a monthly refresher training course and programme-specific training from BRAC (e.g. all SS in the home-fortification programme area receive a 1·5 d training on home fortification of foods with micronutrient powders (MNP)). The BRAC SS maintain a register book containing basic information about their clients such as age, sex, immunization status and status of receiving other health products. They regularly update this register when they visit households in their catchment area and plan their daily home visits based on registered information about their clients.

BRAC provides two type of incentives for SS at the community level, non-financial and financial incentives, to improve their motivation and performance during home visits. Non-financial incentives aim to improve social status. When an SS receives training from BRAC, she becomes a skilled service worker with social identity as a BRAC community health worker. Several BRAC assessments showed that SS enjoy this identity as it gives them importance in their community. BRAC also provides two types of financial incentives to SS. One is for specific services: counselling mothers on infant and young child feeding practices, early initiation of breast-feeding, providing home fortification with MNP, and ensuring compliance and adherence in the treatment/therapy. For example, if an SS ensures breast-feeding within 1 h of birth for a newborn in her catchment area, she receives 50 BDT (Bangladeshi Taka; 1 BDT = $US 0·012). Another is the profit from any products sold by the SS, as SS purchase products from BRAC at a subsidized price and sell them to caregivers with a small profit margin. There are no direct incentives for home visits, but if an SS provides any services or sells any BRAC products to household members she will receive a financial incentive.

**Fig. 1:**
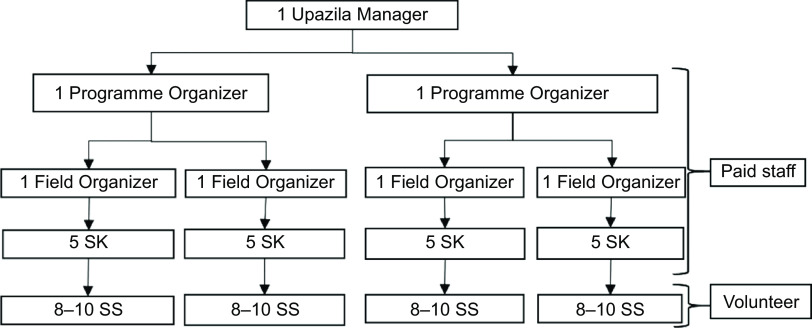
A BRAC service-delivery modality with the various staff members and community health workers (SK, *Sasthya Kormi*; SS, *Shasthya Shebika*) at the sub-district level

In Bangladesh, there are 492 *upazilas* (sub-districts) under sixty-four administrative districts. BRAC’s home-fortification programme is implemented in 164 sub-districts of twenty-six administrative districts (thirty-four BRAC programme districts) in Bangladesh. BRAC splits eight administrative districts into sixteen programme districts. Figure 1 illustrates the organogram of BRAC’s service delivery for a home-fortification programme at the community level. There are three levels of BRAC programmes used to implement the home-fortification interventions: (i) sub-district level; (ii) CHW level; and (iii) community or household level.

1. In every sub-district, BRAC has an office which implements all BRAC programmes. Key staff members at the sub-district level involved in home-fortification programmes are Field Organizers, Programme Organizers and the Upazila Manager, who train BRAC’s CHW (SS and *Sasthya Kormi* (SK)) about home fortification and provide day-to-day monitoring and supervisory supports to SS and SK, inform community members about home-fortification interventions and liaise with community stakeholders.
2. The SS and SK are the lower level of BRAC’s service-delivery platform and implement all BRAC health and nutrition interventions at the community level. The SK, who are paid CHW (by BRAC), supervise the SS. On average eight to ten SS are supervised by one SK (Fig. 1). Both SS and SK are recruited from the local community. In the early 1990s, SS were recruited without considering their academic qualifications. Recently BRAC changed its recruitment criteria: an SS should have at least 5 years of schooling and an SK at least 10 years of schooling.
3. At the community level, the home-fortification intervention is implemented in households with a child aged 6–59 months. BRAC managers at the sub-district level allocate the number of households, ranging from 250 to 450, for each SS.

Since BRAC’s SS are volunteer health workers, they are not accountable for performance of their assigned tasks unlike other paid staff members of BRAC. We observed they had a high dropout rate and low frequency and regularity of home visits^(16–19)^. Irregular home visits by BRAC’s SS have a negative impact on home-fortification coverage and use of MNP products by the caregivers of targeted children^(10)^. However, there is limited research on the role of SS in implementing home-fortification interventions at the community level. The present paper aims to identify factors associated with the number of home visits by SS according to the different levels of BRAC’s programme of home delivery of MNP.

## Methods

### Sources of data

For the current analysis, we considered three sources of data from an evaluation study of BRAC’s home fortification of foods with MNP in Bangladesh. We collected data from the household level, the CHW level and the BRAC programme’s sub-district level, in the sixty-eight sub-districts in the home-fortification programme. We conducted a mixed-methods concurrent evaluation of the programme between 2014 and 2018. It included cross-sectional quantitative surveys and several qualitative investigations, including a process evaluation. For the present paper, we considered the results of the most recent survey which was conducted during February–March 2018 as the source of the household-level data. We also conducted semi-structured interviews with the SS whose catchment area was included in the caregiver survey, which is the source of the CHW-level data. We also collected data on programme staffing and training from all the BRAC sub-districts included in the caregiver survey.

### Survey with community-level caregivers

We conducted survey interviews with caregivers from ten districts using a two-stage clustered sampling strategy. The catchment area of BRAC’s SS was the primary sampling unit. In consultation with programme implementers and experts in home fortification, we selected twenty-two catchment areas of SS from each district through a systematic sampling procedure from the total district list of catchments (range 516–1403 primary sampling units). We calculated a sampling interval by dividing the total number of primary sampling units of each district by the desired number of primary sampling units (twenty-two) from each district and then applied it.

In the second stage of sampling, we identified households through a map segmented–EPI-5 sampling procedure as proposed by the WHO^(20)^. On the day of the interview, the survey team visited the selected catchment areas of the SS and in consultation with the local community leaders, drew a hand-map of the area, divided it into four segments and identified the middle point of each segment. The survey team then randomly selected one segment and, at its geographic centre, spun a bottle to identify the direction from which to count the households. They then selected every fifth household for an interview if the household had an eligible child based on the exclusion and inclusion criteria. If the household did not have an eligible child, the survey team visited the next household to the right of the fifth household. The survey team made three attempts and, if they could not locate an eligible child, they again spun the bottle and followed the same procedures until they found an eligible household. Once an eligible household was found, they spun the bottle to identify the next eligible households. Through this process, the survey team identified two households from the first three segments and one household from the final segment, giving a total of seven.

Within households, the target sample comprised caregivers of children aged 6–59 months in BRAC’s home-fortification programme. We included caregivers who had at least one child aged 6–59 months and selected one child and his/her caregiver from each household. If the eligible household had more than one eligible child and/or caregiver, we randomly selected (by lottery) only one child and his/her caregiver. We defined a caregiver as the individual who had provided most meals to the eligible child in the last 7 d before the survey. We excluded the household if the caregiver reported that he or she was physically and/or mentally unwell and unable to be interviewed; or if the caregiver was absent on the day of interview; or unable to give consent to participate in the survey.

#### Data collection

Survey teams comprising three members, two interviewers and a supervisor, collected the data. The supervisor was mainly responsible for implementing the sampling protocol at the field level, monitoring the data-collection activities and ensuring the quality of data. We used a structured questionnaire that was modelled on the standard questionnaire for evaluating the home fortification of foods in the MNP programme. It asked about sociodemographic variables, home fortification with MNP, interaction with BRAC’s SS and other relevant sections of BRAC’s home-fortification programme. We used an Android-based smartphone program developed by the Information Technology Unit of icddr,b to enter and store data. To support the Android-operating system, we used the Open Data Kit (ODK) software. The tablet/smartphone was used instead of a paper questionnaire (both Bangla and English versions of the questionnaire were used in the ODK software). In addition to the survey interview, we collected and recorded GPS (Global Positioning System) data on caregivers’ households. On average, the interview with caregivers took 35–45 min.

### *Interviews with BRAC’s* Shasthya Shebikas

We conducted a semi-structured interview with BRAC’s SS. We asked all the SS in the sampled catchment areas identified during the first stage of sampling for the caregiver survey to participate. During the survey with caregivers, the survey-team supervisor conducted interviews with the SS at their households separately from the caregiver interviews. We collected a range of information from SS, including their sociodemographic and background characteristics, training received, incentives received, and experiences of supervision and monitoring support. On average, this interview took 10–15 min, including collection of the longitude and latitude of each household covered by the SS.

### Data collection at sub-districts under the BRAC programme

For collecting programme-level data, we identified all the sub-districts containing one or more sampled catchment areas of SS. A checklist form was developed with five programme-level items. We emailed the checklist to the managers of BRAC in all the selected sub-districts and requested them to return the completed form within 4 weeks.

### Measurements

#### Outcomes

The two outcome variables that were considered for the current analysis were: home visits by SS within 12 months of the survey and within 2 months of the survey. Data for these variables were collected at the caregiver level. To estimate indicators of visit recall, we first asked whether the caregiver received any visit by the SS of BRAC in the last 12 months and then asked whether the caregiver received any visit during the 2 months before the survey.

#### Covariates at the household/caregiver (community) level

We selected covariates at different levels which were conceptually linked with home visits by SS of BRAC. We converted all continuous variables into categories because their distribution was not normal, but rather either positively or negatively skewed. We did this based on their median value or the proportioned distribution of categories. The household size of the caregiver was determined based on the number of members in the household, categorized into two groups: 2–4 members and ≥5 members. The age of the child was categorized as 6–23 months and 24–59 months. Children were categorized as male and female, and the age of the caregivers was categorized as ≤23 years and >24 years. The number of children aged 6–59 months living in the household was categorized as 1 child and ≥2 children. The religion of caregivers was categorized with their respective CHW’s religion as: Muslim caregiver and Muslim SS; Muslim caregiver and Hindu (or other religion – Christianity, Buddhism) SS; Hindu (or other) caregiver and Muslim SS; and Hindu (or other) caregiver and Hindu (or other) SS. We recorded educational status as years of schooling both for the caregiver and father of the selected child in the household, and we then categorized them as <5 years and ≥5 years of schooling. The household wealth index was calculated using household materials (e.g. materials used for floor, roof and wall of the house) and household assets (including type of latrine use and sources of drinking-water) and was categorized into three tertiles: poor, middle and rich. We used a nine-item household hunger scale questionnaire to collect data and calculate the food security status of the household, categorized into two broad groups: food-secure and food-insecure household. We considered the child’s morbidity and malnutrition status with an assumption that the child’s health and nutritional status might influence the number of home visits by SS^(3,21)^. We recorded episodes of child’s morbidity: if any morbidity occurred in the 15 d preceding the survey and categorized as ‘yes’ or ‘no’. We recorded the immunization status of children using a proxy indicator of BCG (Bacillus Calmette–Guérin) vaccination as whether or not the child received BCG vaccine. For child’s nutritional status, we assessed whether any child in the household was suffering from acute malnutrition based on the measurement of mid-upper arm circumference (MUAC), categorized as ‘yes’ (MUAC ≤ 12·5 cm) and ‘no’ (MUAC > 12·5 cm). We used our GPS data on the caregivers’ households and SS-covered households to calculate the geospatial distance of the caregiver’s house from the house of their SS, categorized into three groups: distance of caregiver’s household to SS household of 0–299 m, 300–599 m and ≥600 m.

#### Covariates at the *Shasthya Shebika* level

Age of the SS was categorized into three groups (18–30 years; 31–50 years; >50 years). We collected information on completed years of schooling and categorized this into three groups (<5 years, 5–9 years and ≥10 years of schooling). In addition to being female, the other key criterion for the SS to be involved in BRAC’s programme is that they should have ever been married. We categorized marital status as married and other (either widowed or divorced or separated). The main earners in the SS’s household were categorized into three groups: the SS herself as the main earner; the husband of the SS; and other members of the household (e.g. father or brother of the SS). We recorded monthly income in Bangladeshi Taka (1 BDT = $US 0·012) as a continuous variable and categorized it as: ≤7000 BDT, 7001–12 000 BDT and >12 000 BDT. The amount of incentives received by the SS in the last 3 months prior to data collection was categorized into four groups: <100 BDT, 100–400 BDT, 401–800 BDT and >800 BDT, considering the proportion of SS available in each of the groups. We asked whether the SS received programme-specific basic training and whether they received any monitoring visit from other BRAC staff in the last 3 months before the survey; for both of these questions, the response was either ‘yes’ or ‘no’. The length of work was recorded in complete years of working experience as an SS and categorized into two groups: 1–6 years and >6 years.

#### Covariates of BRAC’s programme at the sub-district level

We recorded the number of SS available in each sub-district for implementing the BRAC’s nutrition programme and categorized it into three groups: ≤99 SS, 100–199 SS and >200 SS. At the sub-district level, the SS were supposed to receive at least one refresher training course every month. We collected this information about the number of refresher training courses received by each SS within 6 months of the survey in the sub-district and categorized it into three groups: <4, 4–7 and >7. The availability of supervisory staff members at the sub-district level was categorized as ≤2 and >2. We also recorded whether there was any SS vacancy at the sub-district level during data collection; the response was recorded as ‘yes’ or ‘no’.

### Statistical methods

The statistical software package Stata version 15 was used for analysing data. We used the survey commands and weighted the data to adjust for disproportionate sampling (cluster sampling) and non-response. We used frequencies and percentages to describe different characteristics of the caregivers, SS and BRAC’s programme at the sub-district level. As the caregivers were nested within the catchment areas of SS and the catchment areas of SS were nested within sub-districts, we performed a multilevel logistic regression to estimate the effect of factors relating to the households, SS and BRAC’s programme at the sub-district level on the home visits of SS within 12 months and 2 months of the survey. We captured the correlation at different levels by performing multilevel modelling.

We used the *xtmelogit* command of the Stata software to run the multilevel random-intercept logistic regression model and ran four models. First, we ran the empty model (Model 0) or null model, which contained no covariates, to calculate the intracluster correlation to measure the variance in home contact among the SS across the BRAC programme at the sub-district level. This model helped us assess the level of correlation between clusters within a model and to compare the successive models by looking at the decline of the intracluster correlation. For example, in the null model, intracluster correlation was 0·20; it decreased to 0·17 in Model 1, 0·12 in Model 2 and 0·09 in Model 3 for SS visit within 12 months of the survey. Model 1 contained the household/caregiver-level covariates only. Model 2 contained the SS-level covariates in addition to the household/caregiver-level covariates. Model 3 is the full model with the combined covariates from the household/caregiver level, SS level and BRAC’s programme at the sub-district level. We calculated odds ratios and adjusted odds ratios (AOR) to measure the association between the household/caregiver-, SS- and sub-district-level factors on the home visits by the SS and used 95 % confidence intervals to estimate the population effect sizes. Our analyses only included sub-districts where data were available and could be linked for all three levels (household/caregiver, SS, sub-district).

The sample size was based on the requirements for evaluation of the MNP home-fortification programme from which the data were obtained. A sample size of 1120 households was required to detect a 10 % reduction in the prevalence of anaemia (the primary outcome for the evaluation) from the baseline to endline survey. For the multilevel analysis, *post hoc* calculations indicated that this sample size would allow for detection of a difference in binary characteristics of approximately 12 % for households with and without an SS visit within the past 12 months and 15 % for households with and without an SS visit in the past 2 months, assuming 80 % power, 5 % significance level, a prevalence of SS visits within the previous 12 months of 50 % and within the previous 2 months of 25 %, and a design effect of 2.

## Results

Our analyses included data from 1408 caregivers (out of 1540 caregivers) and 201 SS (out of 220 SS) from fifty-four sub-districts under BRAC’s programme (out of sixty-eight sub-districts). Table 1 shows the socioeconomic, demographic and other background characteristics of the study participants and BRAC’s programmes at the sub-district level. Almost half (*n* 692, 49 %) of the households were visited by an SS within the past 12 months and 330 (23 %) had a visit within the 2 months prior to the survey. Table 2 shows the associations between the two outcomes and the covariates at the household or caregiver level, SS level and the sub-district level. First, we present details of the number and percentage of households that received a home visit by the SS within 12 months and 2 months of the survey under different categories of the covariates. We then present the results of the logistic regression with unadjusted odds ratios. In the unadjusted model, households with younger children, with higher education of caregivers and having higher wealth index were significantly associated with higher odds of receiving home visits by an SS. The odds of home visits decreased with increasing distance between the caregiver’s house and their SS’s house. At the SS level, households received more visits if the SS received more incentives, were the main household income-earner and received more monitoring visits from their supervisors. None of the sub-district-level variables were significantly associated with SS’s home visits (Table 2).

**Table 1:** Socio-economic, demographic and other background characteristics of households or caregivers at the community level, *Shasthya Shebikas* (SS) and BRAC’s programme at the sub-district level†

Variable	*n*	%
Covariates at the household/caregiver level (*n* 1408)		
Household size (number of household members)		
2–4	619	44·0
≥5	789	56·0
Number of children aged 6–59 months in household		
1 child	1254	89·1
≥2 children	154	10·9
Wealth index of the households		
Poor	470	33·4
Middle	480	34·1
Rich	458	32·5
Food security status of household		
Secure	948	67·3
Insecure	460	32·7
Distance of caregiver’s house from SS’s house		
0–299 m	738	56·8
300–599 m	410	31·6
≥600 m	151	11·7
Home visit by SS to caregiver’s households		
Home visit within 12 months	692	49·2
Home visit within 2 months	330	23·4
Religion (categorized with SS’s religion)		
Muslim caregiver and Muslim SS	1224	86·9
Hindu (with other) caregiver and Muslim SS	58	4·1
Muslim caregiver and Hindu SS	77	5·5
Hindu (with other) caregiver and Hindu SS	49	3·5
Caregiver’s age		
≤23 years	474	33·7
>24 years	934	66·3
Caregiver’s education (completed years of schooling)		
≥5 years	1031	73·2
<5 years	377	26·8
Father’s education (completed years of schooling)		
≥5 years	861	61·2
<5 years	547	38·9
Child’s age		
6–23 months	560	39·8
24–59 months	848	60·2
Child’s sex		
Male	728	51·7
Female	680	48·3
Child’s illness incidence (morbidity) in past 14 d		
Yes	685	48·7
No	723	51·4
Children in household suffering from undernutrition		
No (MUAC ≥ 12·5 cm)	1357	96·5
Yes (MUAC < 12·5 cm)	50	3·6
Child’s vaccination coverage (BCG vaccination)		
No	243	17·2
Yes	1165	82·7
Covariates at the SS level (*n* 201)		
Age		
18–30 years	25	12·4
31–50 years	120	59·7
>50 years	56	27·9
SS education (completed years of schooling)		
<5 years	94	46·8
5–9 years	97	48·3
≥10 years	10	5·0
Marital status		
Married	148	73·6
Other (e.g. widowed/separated)	53	26·4
Amount of incentive received in last 3 months as SS		
<100 BDT	24	12·3
100–400 BDT	60	30·8
401–800 BDT	71	36·4
>800 BDT	40	20·5
Received basic training from BRAC		
No	16	8·0
Yes	185	92·0
Received monitoring visit from other BRAC staff		
No	109	54·2
Yes	92	45·8
Main earner of SS’s household		
Other member (e.g. SS’s father/son)	54	26·9
Husband of SS	121	60·2
SS herself	26	12·9
Length of working as SS		
1–6 years	64	31·8
>6 years	137	68·2
Monthly income of SS’s household		
≤7000 BDT	70	35·4
7001–12 000 BDT	68	34·3
>12 000 BDT	60	30·3
Covariates at the programme level at the sub-district (*n* 54)		
Number of SS available in BRAC’s programme at the sub-districts		
≤99	9	16·7
100–199	31	57·4
≥200	14	25·9
Availability of supervisory staff members		
≤2	47	87·0
>2	7	13·0
Any vacancy of staff at the sub-district level		
No	23	42·6
Yes	31	57·4
Number of refresher trainings received by SS		
<4	11	20·4
4–7	23	42·6
>7	20	37·0

MUAC, mid-upper arm circumference; BCG, Bacillus Calmette–Guérin; BDT, Bangladeshi Taka.

Data are from an evaluation study of BRAC’s home fortification of foods with micronutrient powders in sixty-eight sub-districts in Bangladesh, 2014–2018.

† Data are adjusted for the sampling design.

**Table 2:** Associations between home contact with *Shasthya Shebikas* (SS) and independent variables at the household/caregiver level, SS level and programme level

Variable	Outcome							
	Home contact in the last 12 months (*n* 692; 49 %)				Home contact in the last 2 months (*n* 330; 23 %)			
	*n*	%	OR	95 % CI	*n*	%	OR	95 % CI
Covariates at the household/caregiver level								
Household size (number of household members)								
2–4	300	43·4	1·00	Ref.	144	43·6	1·00	Ref.
≥5	392	56·7	1·01	0·80, 1·29	186	56·4	0·94	0·71, 1·25
Child’s age								
6–23 months	318	46·0	1·00	Ref.	158	47·9	1·00	Ref.
24–59 months	374	54·1	0·56****	0·44, 0·72	172	52·1	0·63***	0·48, 0·83
Child’s sex								
Male	368	53·2	1·00	Ref.	176	53·3	1·00	Ref.
Female	324	46·8	0·89	0·70, 1·12	154	46·7	0·96	0·73, 1·26
Caregiver’s age								
≤23 years	228	33·0	1·00	Ref.	106	32·1	1·00	Ref.
>24 years	464	67·1	1·09	0·85, 1·40	224	67·9	1·20	0·89, 1·62
Number of children aged 6–59 months in household								
1 child	611	88·3	1·00	Ref.	290	87·9	1·00	Ref.
≥2 children	81	11·7	1·13	0·77, 1·65	40	12·1	1·14	0·74, 1·76
Caregiver’s education (completed years of schooling)								
<5 years	155	22·4	1·00	Ref.	70	21·2	1·00	Ref.
≥5 years	537	77·6	1·53***	1·16, 2·00	260	78·8	1·51**	1·09, 2·11
Father’s education (completed years of schooling)								
<5 years	251	36·3	1·00	Ref.	123	37·3	1·00	Ref.
≥5 years	441	63·7	1·14	0·88, 1·45	207	62·7	1·02	0·76, 1·36
Wealth index								
Poor	211	30·5	1·00	Ref.	108	32·7	1·00	Ref.
Middle	250	36·1	1·34*	1·00, 1·79	122	37·0	1·13	0·80, 1·58
Rich	231	33·4	1·26	0·93, 1·70	100	30·3	0·87	0·61, 1·24
Food security status of household								
Secure	463	66·9	1·00	Ref.	225	68·2	1·00	Ref.
Insecure	229	33·1	1·03	0·79, 1·33	105	31·8	0·88	0·65, 1·19
Child’s illness incidence (morbidity) in past 14 d								
No	349	50·4	1·00	Ref.	170	51·5	1·00	Ref.
Yes	343	49·6	1·16	0·91, 1·48	160	48·5	1·01	0·76, 1·34
Children in household suffering from undernutrition								
No (MUAC ≥ 12·5 cm)	665	96·1	1·00	Ref.	320	97·0	1·00	Ref.
Yes (MUAC < 12·5 cm)	27	3·9	1·15	0·61, 2·19	10	3·0	0·77	0·35, 1·69
Child’s vaccination coverage (BCG vaccination)								
No	115	16·6	1·00	Ref.	46	13·9	1·00	Ref.
Yes	577	83·4	1·13	0·82, 1·56	284	86·1	1·52**	1·02, 2·26
Distance of caregiver’s house from SS’s house								
0–299 m	383	60·3	1·00	Ref.	184	60·9	1·00	Ref.
300–599 m	188	29·6	0·74**	0·56, 0·99	91	30·1	0·78	0·56, 1·09
≥600 m	64	10·1	0·67*	0·44, 1·03	27	8·9	0·59*	0·35, 1·01
Religion (categorized with SS’s religion)								
Muslim caregiver and Muslim SS	584	84·4	1·00	Ref.	277	83·9	1·00	Ref.
Hindu (with other) caregiver and Muslim SS	38	5·5	1·74*	0·90, 3·34	22	6·7	1·94*	0·99, 3·83
Muslim caregiver and Hindu SS	38	5·5	1·10	0·57, 2·14	12	3·6	0·65	0·28, 1·51
Hindu (with other) caregiver and Hindu SS	32	4·6	2·51**	1·11, 5·67	19	5·8	2·46**	1·05, 5·77
Covariates at the SS level								
Age								
18–30 years	87	12·6	1·00	Ref.	41	12·4	1·00	Ref.
31–50 years	432	62·4	1·07	0·64, 1·77	208	63·0	1·04	0·57, 1·90
>50 years	173	25·0	0·77	0·44, 1·35	81	24·6	0·83	0·43, 1·60
SS’s education (completed years of schooling)								
<5 years	310	44·8	1·00	Ref.	163	49·4	1·00	Ref.
5–9 years	347	50·1	1·04	0·74, 1·47	153	46·4	0·77	0·52, 1·15
≥10 years	35	5·1	1·06	0·49, 2·26	14	4·2	0·72	0·29, 1·82
Marital status								
Married	175	25·3	1·00	Ref.	81	24·6	1·00	Ref.
Other (e.g. widowed/separated)	517	74·7	1·16	0·79, 1·71	249	75·5	1·21	0·77, 1·89
Amount of incentive received in last 3 months as SS								
<100 BDT	58	8·6	1·00	Ref.	15	4·7	1·00	Ref.
100–400 BDT	185	27·4	1·46	0·85, 2·53	90	28·0	3·01***	1·43, 6·36
401–800 BDT	259	38·4	2·05**	1·19, 3·54	120	37·3	3·54***	1·70, 7·38
>800 BDT	173	25·6	3·10****	1·69, 5·69	97	30·1	6·13****	2·80, 13·42
Received basic training from BRAC								
No	52	7·5	1·00	Ref.	17	5·2	1·00	Ref.
Yes	640	92·5	1·05	0·56, 1·95	313	94·9	1·95*	0·89, 4·26
Received monitoring visit from other BRAC staff								
No	345	49·9	1·00	Ref.	151	45·8	1·00	Ref.
Yes	347	50·1	1·48**	1·07, 2·06	179	54·2	1·67***	1·14, 2·43
Main earner of SS’s household								
Other member (e.g. SS’s father/son)	165	23·8	1·00	Ref.	67	20·3	1·00	Ref.
Husband of SS	432	62·4	1·30	0·89, 1·89	210	63·6	1·53*	0·97, 2·39
SS herself	95	13·8	1·36	0·79, 2·34	53	16·1	2·06**	1·10, 3·84
Length of working as SS								
1–6 years	233	33·7	1·00	Ref.	104	31·5	1·00	Ref.
>6 years	459	66·3	0·81	0·57, 1·15	226	68·5	1·04	0·69, 1·57
Monthly income of SS’s household								
≤7000 BDT	243	35·4	1·00	Ref.	125	38·3	1·00	Ref.
7001–12 000 BDT	231	33·7	0·83	0·56, 1·24	99	30·4	0·65*	0·40, 1·05
>12 000 BDT	212	30·9	0·93	0·62, 1·41	102	31·3	0·87	0·54, 1·40
Covariates at the programme level								
Number of SS available in BRAC’s programme at the sub-districts								
≤99	58	8·4	1·00	Ref.	29	8·8	1·00	Ref.
100–199	372	53·8	1·66	0·85, 3·23	163	49·4	1·18	0·57, 2·43
≥200	262	37·9	1·81	0·89, 3·68	138	41·8	1·63	0·76, 3·46
Availability of supervisory staff members								
≤2	585	84·5	1·00	Ref.	278	84·2	1·00	Ref.
>2	107	15·5	1·31	0·70, 2·45	52	15·8	1·21	0·65, 2·26
Any vacancy of staff at sub-district level								
No	248	35·8	1·00	Ref.	113	34·2	1·00	Ref.
Yes	444	64·2	0·91	0·57, 1·45	217	65·8	1·08	0·66, 1·75
Number of refresher training courses received by SS								
<4	127	18·4	1·00	Ref.	58	17·6	1·00	Ref.
4–7	317	45·8	1·54	0·89, 2·67	148	44·9	1·41	0·80, 2·50
>7	248	35·8	1·72*	0·95, 3·12	124	37·6	1·74*	0·94, 3·21

MUAC, mid-upper arm circumference; BCG, Bacillus Calmette–Guérin; BDT, Bangladeshi Taka; ref., reference category.

Data are from an evaluation study of BRAC’s home fortification of foods with micronutrient powders in sixty-eight sub-districts in Bangladesh, 2014–2018.

*P < 0·1, **P < 0·05, ***P < 0·01, ****P < 0·001.

Table 3 presents the results of multilevel modelling for the outcome of a home visit by the SS within 12 months of the survey and 2 months of the survey. In the full model, households with older children aged 24–59 months, compared with younger children, had significantly lower odds of an SS visit in the preceding 12 months (AOR = 0·55; 95 % CI 0·42, 0·72; *P* < 0·001) and 2 months (AOR = 0·62; 95 % CI 0·46, 0·85; *P* = 0·003). The AOR for home visits within 12 months by the SS was 1·53 (95 % CI 1·10, 2·12; *P* = 0·011) and within 2 months was 1·58 (95 % CI 1·06, 2·35; *P* = 0·025) in households where the caregiver had ≥5 years of education. Increasing distance between the caregiver’s house and the SS’s house was associated with reduced odds of an SS visit with 12 months (AOR = 0·67; 95 % CI 0·50, 0·89; *P* = 0·006 for 300–599 m and AOR = 0·64; 95 % CI 0·42, 1·00; *P* = 0·047 for ≥600 m, relative to 0–299 m) and within 2 months (AOR = 0·68; 95 % CI 0·48, 0·97; *P* = 0·032 and AOR = 0·56; 95 % CI 0·32, 0·98; *P* = 0·042 for 300–599 and ≥600 m, respectively). The highest wealth index tertile was associated with lower odds of an SS visit within 2 months, and child vaccination coverage was associated with almost twofold increased odds of a visit. There was some indication of an association between religion and SS visits, but this was only statistically significant at the 5 % level for an SS visit within 12 months when there was a Hindu caregiver and Hindu SS, with an AOR of approximately 2·5.

**Table 3:** Results of multilevel modelling for home visits by *Shasthya Shebikas* (SS) within 12 and 2 months of the survey

Covariate	Home visit within 12 months of the survey						Home visit within 2 months of the survey					
	Model 1		Model 2		Model 3		Model 1		Model 2		Model 3	
	AOR	95 % CI	AOR	95 % CI	AOR	95 % CI	AOR	95 % CI	AOR	95 % CI	AOR	95 % CI
Covariates at the household/caregiver level												
Household size (number of household members)												
2–4	1·00	Ref.	1·00	Ref.	1·00	Ref.	1·00	Ref.	1·00	Ref.	1·00	Ref.
≥5	1·09	0·85, 1·40	1·16	0·90, 1·50	1·18	0·91, 1·45	0·98	0·72, 1·33	1·03	0·76, 1·40	1·05	0·77, 1·43
Child’s age												
6–23 months	1·00	Ref.	1·00	Ref.	1·00	Ref.	1·00	Ref.	1·00	Ref.	1·00	Ref.
24–59 months	0·55****	0·43, 0·72	0·54****	0·42, 0·72	0·55***	0·42, 0·72	0·62***	0·46, 0·84	0·62***	0·46, 0·85	0·62***	0·46, 0·85
Child’s sex												
Male	1·00	Ref.	1·00	Ref.	1·00	Ref.	1·00	Ref.	1·00	Ref.	1·00	Ref.
Female	0·94	0·74, 1·21	0·97	0·75, 1·25	0·96	0·75, 1·24	1·01	0·75, 1·35	1·05	0·78, 1·42	1·05	0·78, 1·42
Caregiver’s age												
≤23 years	1·00	Ref.	1·00	Ref.	1·00	Ref.	1·00	Ref.	1·00	Ref.	1·00	Ref.
>24 years	1·22	0·93, 1·61	1·21	0·92, 1·60	1·19	0·90, 1·57	1·35*	0·97, 1·87	1·26	0·91, 1·75	1·25	0·90, 1·74
Number of children aged 6–59 months in household												
1 child	1·00	Ref.	1·00	Ref.	1·00	Ref.	1·00	Ref.	1·00	Ref.	1·00	Ref.
≥2 children	1·03	0·68, 1·56	1·03	0·67, 1·58	1·03	0·67, 1·57	1·18	0·73, 1·91	1·19	0·72, 1·97	1·20	0·73, 2·00
Caregiver’s education (completed years of schooling)												
<5 years	1·00	Ref.	1·00	Ref.	1·00	Ref.	1·00	Ref.	1·00	Ref.	1·00	Ref.
≥5 years	1·54***	1·12, 2·12	1·56***	1·12, 2·16	1·53**	1·10, 2·12	1·61*	1·09, 2·38	1·59**	1·07, 2·37	1·58**	1·06, 2·35
Father’s education (completed years of schooling)												
<5 years	1·00	Ref.	1·00	Ref.	1·00	Ref.	1·00	Ref.	1·00	Ref.	1·00	Ref.
≥5 years	0·96	0·71, 1·29	0·95	0·69, 1·27	0·96	0·71, 1·30	0·98	0·69, 1·39	1·00	0·70, 1·43	1·02	0·71, 1·46
Wealth index												
Poor	1·00	Ref.	1·00	Ref.	1·00	Ref.	1·00	Ref.	1·00	Ref.	1·00	Ref.
Middle	1·35*	0·98, 1·86	1·30	0·94, 1·80	1·30	0·94, 1·81	1·04	0·71, 1·51	0·97	0·66, 1·42	0·96	0·65, 1·41
Rich	1·13	0·79, 1·61	1·02	0·71, 1·48	1·04	0·72, 1·50	0·71	0·46, 1·09	0·63**	0·41, 0·98	0·63**	0·41, 0·99
Food security status of household												
Secure	1·00	Ref.	1·00	Ref.	1·00	Ref.	1·00	Ref.	1·00	Ref.	1·00	Ref.
Insecure	1·23	0·91, 1·66	1·16	0·85, 1·58	1·14	0·84, 1·56	0·91	0·63, 1·30	0·86	0·59, 1·24	0·86	0·59, 1·25
Child’s illness incidence (morbidity) in past 14 d												
Yes	1·00	Ref.	1·00	Ref.	1·00	Ref.	1·00	Ref.	1·00	Ref.	1·00	Ref.
No	1·10	0·86, 1·42	1·04	0·80, 1·35	1·04	0·80, 1·35	1·00	0·74, 1·34	0·96	0·70, 1·30	0·96	0·71, 1·30
Children in household suffering from undernutrition												
No (MUAC ≥ 12·5 cm)	1·00	Ref.	1·00	Ref.	1·00	Ref.	1·00	Ref.	1·00	Ref.	1·00	Ref.
Yes (MUAC < 12·5 cm)	1·08	0·55, 1·42	1·03	0·51, 2·06	1·02	0·51, 2·05	0·72	0·31, 1·63	0·62	0·27, 1·47	0·62	0·26, 1·45
Child’s vaccination coverage (BCG vaccination)												
No	1·00	Ref.	1·00	Ref.	1·00	Ref.	1·00	Ref.	1·00	Ref.	1·00	Ref.
Yes	1·22	0·88, 1·71	1·16	0·82, 1·64	1·14	0·81, 1·61	1·87**	1·21, 2·90	1·91***	1·22, 3·00	1·91***	1·21, 2·99
Distance of caregiver’s house from SS’s house												
0–299 m	1·00	Ref.	1·00	Ref.	1·00	Ref.	1·00	Ref.	1·00	Ref.	1·00	Ref.
300–599 m	0·71**	0·53, 0·95	0·68***	0·51, 0·91	0·67***	0·50, 0·89	0·74*	0·52, 1·04	0·68**	0·48, 0·97	0·68**	0·48, 0·97
≥600 m	0·67*	0·43, 1·04	0·64**	0·41, 1·00	0·64**	0·42, 1·00	0·57*	0·33, 1·01	0·54**	0·31, 0·95	0·56**	0·32, 0·98
Religion (categorized with SS’s religion)												
Muslim caregiver and Muslim SS	1·00	Ref.	1·00	Ref.	1·00	Ref.	1·00	Ref.	1·00	Ref.	1·00	Ref.
Hindu (with other) caregiver and Muslim SS	1·77*	0·90, 3·49	1·70	0·86, 3·38	1·79*	0·91, 3·52	2·22**	1·10, 4·51	1·96*	0·97, 3·97	2·01*	0·99, 4·08
Muslim caregiver and Hindu SS	1·19	0·62, 2·29	1·16	0·61, 2·21	1·33	0·70, 2·52	0·70	0·30, 1·66	0·64	0·28, 1·46	0·68	0·30, 1·58
Hindu (with other) caregiver and Hindu SS	2·45**	1·10, 5·44	2·20*	0·95, 5·09	2·52*	1·09, 5·79	2·45**	1·04, 5·79	1·89	0·80, 4·48	2·07*	0·87, 4·91
Covariates at the SS level												
Age												
18–30 years	–	–	1·00	Ref.	1·00	Ref.	–	–	1·00	Ref.	1·00	Ref.
31–50 years	–	–	0·85	0·51, 1·45	0·73	0·43, 1·23	–	–	0·67	0·36, 1·24	0·64	0·34, 1·19
>50 years	–	–	0·62	0·35, 1·13	0·57*	0·32, 1·03	–	–	0·45**	0·22, 0·91	0·44**	0·21, 0·90
SS education (completed years of schooling)												
<5 years	–	–	1·00	Ref.	1·00	Ref.	–	–	1·00	Ref.	1·00	Ref.
5–9 years	–	–	1·04	0·72, 1·48	1·12	0·78, 1·61	–	–	0·74	0·49, 1·14	0·80	0·50, 1·18
≥10 years	–	–	0·95	0·45, 2·03	0·89	0·42, 1·65	–	–	0·61	0·24, 1·55	0·58	0·23, 1·47
Marital status												
Married	–	–	1·00	Ref.	1·00	Ref.	–	–	1·00	Ref.	1·00	Ref.
Other (e.g. widowed/separated)	–	–	0·98	0·61, 1·58	1·03	0·65, 1·65	–	–	1·29	0·72, 2·31	1·30	0·72, 1·47
Amount of incentive received in last 3 months as SS												
<100 BDT	–	–	1·00	Ref.	1·00	Ref.	–	–	1·00	Ref.	1·00	Ref.
100–400 BDT	–	–	1·27	0·73, 2·20	1·49	0·85, 2·58	–	–	2·73**	1·27, 5·84	2·86***	1·32, 6·20
401–800 BDT	–	–	1·94**	1·13, 3·32	2·10***	1·23, 3·58	–	–	3·02***	1·42, 6·39	3·17***	1·48, 6·80
>800 BDT	–	–	2·59***	1·39, 4·83	3·00***	1·58, 5·58	–	–	4·87****	2·13, 11·09	5·36****	2·28, 12·56
Received basic training from BRAC												
No	–	–	1·00	Ref.	1·00	Ref.	–	–	1·00	Ref.	1·00	Ref.
Yes	–	–	0·64	0·32, 1·26	0·66	0·34, 1·27	–	–	1·13	0·48, 2·69	1·15	0·49, 2·70
Received monitoring visit from other BRAC staff												
No	–	–	1·00	Ref.	1·00	Ref.	–	–	1·00	Ref.	1·00	Ref.
Yes	–	–	1·29	0·93, 1·79	1·28	0·93, 1·78	–	–	1·48**	1·00, 2·19	1·48*	1·00, 2·19
Main earner of SS’s household												
Other member (e.g. SS’s father/son)			1·00	Ref.	1·00	Ref.			1·00	Ref.	1·00	Ref.
Husband of SS			1·13	0·74, 1·79	1·11	0·73, 1·69			1·36	0·81, 2·31	1·31	0·78, 2·21
SS herself			1·12	0·59, 2·15	1·26	0·66, 2·40			2·19**	1·02, 4·67	2·14**	1·00, 4·58
Length of working as SS												
1–6 years	–	–	1·00	Ref.	1·00	Ref.	–	–	1·00	Ref.	1·00	Ref.
>6 years	–	–	1·15	0·78, 1·70	1·25	0·85, 1·85	–	–	1·17	0·74, 1·85	1·16	0·73, 1·85
Monthly income of SS’s household												
≤7000 BDT	–	–	1·00	Ref.	1·00	Ref.	–	–	1·00	Ref.	1·00	Ref.
7001–12 000 BDT	–	–	0·71	0·44, 1·15	0·74	0·46, 1·19	–	–	0·76	0·44, 1·32	0·72	0·41, 1·24
>12 000 BDT	–	–	0·82	0·52, 1·29	0·82	0·52, 1·27	–	–	0·91	0·54, 1·53	0·86	0·51, 1·45
Covariates at the programme level												
Number of SS available in BRAC’s programme at the sub-districts												
≤99	–	–	–	–	1·00	Ref.	–	–	–	–	1·00	Ref.
100–199	–	–	–	–	2·83***	1·50, 5·33	–	–	–	–	1·81	0·79, 4·11
≥200	–	–	–	–	3·15***	1·65, 6·04	–	–	–	–	2·28*	0·97, 5·38
Availability of supervisory staff members												
≤2	–	–	–	–	1·00	Ref.	–	–	–	–	1·00	Ref.
>2	–	–	–	–	1·04	0·64, 1·70	–	–	–	–	1·02	0·53, 1·96
Any vacancy of staff at sub-district level												
No	–	–	–	–	1·00	Ref.	–	–	–	–	1·00	Ref.
Yes	–	–	–	–	1·04	0·46, 1·02	–	–	–	–	0·90	0·53, 1·52
Number of refresher training courses received by SS												
<4	–	–	–	–	1·00	Ref.	–	–	–	–	1·00	Ref.
4–7	–	–	–	–	1·22	0·76, 1·96	–	–	–	–	0·98	0·51, 1·87
>7	–	–	–	–	1·41*	0·86, 2·31	–	–	–	–	1·04	0·54, 2·00

AOR, adjusted odds ratio; MUAC, mid-upper arm circumference; BCG, Bacillus Calmette–Guérin; BDT, Bangladeshi Taka; ref., reference category.

Data are from an evaluation study of BRAC’s home fortification of foods with micronutrient powders in sixty-eight sub-districts in Bangladesh, 2014–2018.

Model 1 considered household/caregiver-level covariates. Model 2 considered household/caregiver- and SS-level covariates. Model 3 considered household/caregiver-, SS- and programme-level covariates.

*P < 0·10, **P < 0·05, ***P < 0·01, ****P < 0·001.

Increased incentives were significantly associated with home visit by the SS within both 12 months and 2 months of the survey. In the full model, the AOR for home visits by the SS within 12 months was 2·10 (95 % CI 1·23, 3·58; *P* = 0·006) and the AOR was 3·17 (95 % CI 1·48, 6·80; *P* = 0·003) for home visits within 2 months of the survey in the households in the catchment areas of SS who received between 400 and 800 BDT as an incentive. The AOR were even higher for incentives of >800 BDT: AOR = 3·00 (95 % CI 1·58, 5·58; *P* = 0·001) and AOR = 5·36 (95 % CI 2·28, 12·56; *P* < 0·001) for an SS visit within 12 months and 2 months, respectively. In the full model, the age of SS was significantly associated with the home visits by SS within the last 2 months: AOR = 0·44 (95 % CI 0·21, 0·90; *P* = 0·025) for the households with SS who were aged >50 years compared with the SS aged 18–30 years (Table 3). The AOR for a home visit within 2 months was 2·14 (95 % CI 1·00, 4·58; *P* = 0·049) in areas where the SS were the main earners (Table 3). At the sub-district level, if the BRAC programme had more than 100 SS, households in those sub-districts had approximately three times the odds of an SS visit within 12 months compared with sub-districts with less than 99 SS (Table 3). There was indication of an association between higher odds of SS visits within 12 months and being in a sub-district where SS received more than seven refresher training courses, and higher odds of an SS visit within 2 months and an SS monitoring visit; however, these were significant only at the 10 %, not 5 %, level (Table 3).

## Discussion

Our study identified a number of factors at the household level which were significantly associated with home visits by an SS to provide BRAC’s home-fortification services. Most health and nutrition services provided by SS at the community level target pregnant women and younger children aged <2 years^(13,22)^. Results of our analysis suggest that households with younger children are more likely to be visited by an SS than households with older children. As younger children in households are perceived to be more vulnerable compared with older ones, households with younger children usually have higher demand for services from the CHW. In Bangladesh, a number of successful health programmes for younger children have been implemented using CHW^(7,23–25)^.

The CHW in low-income settings generally find it easy to interact with caregivers who are educated or are better able to understand their health messages and to convert these messages into action^(24)^. For example, earlier studies^(26,27)^ found that caregivers with more education are better able to understand the nutrition-related messages compared with caregivers who have no or limited education. Our study observed that the households of caregivers with more than 5 years of completed schooling received more home visits by the SS compared with households with caregivers who completed less than 5 years of schooling. The ability of educated caregivers potentially helped CHW achieve their performance targets because they are more likely to understand the nutrition messages, purchase the product and use the product with better adherence. Our study also demonstrated that households in catchment areas received more home visits from the SS who received more incentives. The SS might receive higher incentives because visiting more homes created the opportunity to sell more products or because she provided more services that attracted incentives. This finding also corroborates the findings of a study that financial incentives for BRAC’s SS are the key to success of its health and nutrition programme at the community level^(18)^.

The geographical distance between the households of caregivers and those of their respective CHW is a critical factor in the effective performance of CHW^(28)^. Our study observed that if the distance increased, the odds of home visits by SS decreased significantly. Caregivers who reside close to their CHW may have a better social relationship with them. This would build confidence to provide and receive services. Better understanding of the CHW by caregivers is likely to help build trust in the CHW’s services which will contribute to the success of nutrition interventions at the community level^(10,11)^. Furthermore, CHW need to spend more time and energy to reach households that are further away and this may present an additional difficulty, particularly for older CHW who have a lower odds of home visits compared with younger SS.

It is important to distribute the targeted households carefully among the SS. As volunteer health workers, they will be expected to complete their own household activities before visiting other households. This may be difficult if their targeted households are scattered far from their own households. One possible solution may be recruiting an adequate number of SS and distributing their targeted households considering the proximity. On the other hand, ensuring the availability of more SS and retaining them in the community activities are very critical^(17,29)^ as the availability of more SS in the sub-district was associated with increased odds of household visits^(30)^.

Supportive supervision and regular monitoring of CHW may be positively associated with more home visits^(4,21,31)^. There was an indication that there were higher odds of SS making visits when they were monitored by their supervisory staff, although this association was significant only at the 10 % level. Through monitoring visits, supervisors may encourage CHW to make more visits including to more distant homes and create an opportunity for CHW to discuss the challenges they face for promoting their services in the communities. BRAC SS reported feeling comfortable when they visited communities with their immediate supervisor, that is SK^(29)^.

The present study can make a significant policy contribution by filling in the potential gaps in BRAC’s service-delivery network and providing an opportunity for BRAC to improve its CHW model. In Bangladesh, BRAC has played an important role in implementing a number of large-scale nutrition programmes and assisting the Ministry of Health and Family Welfare in addressing nutrition challenges. Such a contribution makes BRAC a major partner of the Ministry of Health and Family Welfare in Bangladesh. The National Nutrition Services under the Ministry of Health and Family Welfare does not have health workers to provide nutrition services at the community level. In such a situation, BRAC’s SS played a critical role in filling this service-delivery gap in the country’s health systems. Through regular home visits, the SS can implement a home-fortification programme which potentially contributes to achieving the country’s health and nutrition-related Sustainable Development Goals.

Our study has several limitations. In the evaluation study we could only link data for three levels: household, SS and BRAC sub-district programme, for fifty-four of the sixty-eight sub-districts, which contained twenty-two BRAC communities. Therefore, the number of home visits we observed in the present study may not be representative of other districts and communities. In Bangladesh, two types of CHW work in communities (e.g. paid and volunteer); whereas, in the present study, we captured information only about unpaid SS who are volunteer CHW. Therefore, the performance of BRAC’s SS may not be comparable with that of other CHW funded from other sources. At present we do not have capacity to unpick the direction of some relationships between SS home visits and other factors. For example, we cannot tell if incentives encourage SS to make home visits to perform the activities that attract incentives or whether SS who pay more visits for other reasons, such as being younger or closer, receive more incentives. We also do not know whether SS visit households in response to transitory illnesses or only respond to these conditions if they occur when they perform their regular visits. A strength is that the large data set and availability of information at the different levels of implementation enable multilevel analysis for a greater understanding of the complex interplay of elements in the BRAC programme.

## Conclusions

We have demonstrated that the number and regularity of home visits are a function of the characteristics of SS, factors that characterize the households they serve and characteristics of their organizational context, particularly to implement BRAC’s home-fortification of foods with MNP. It is important for BRAC to consider these factors to increase regular home visits by the SS. BRAC also could revisit the SS model in order to provide better financial support for SS which might enable them to make more visits to targeted households. Moreover, to address distance-related challenges, BRAC should carefully allocate the households so that an SS can reach all households comfortably and, if there is any hard-to-reach household, BRAC may allocate hard-to-reach allowances and reimbursement of transportation cost if there is any.
